# Concurrent Presentation of Eczema Herpeticum and Acute Localized Exanthematous Pustulosis in a Patient With Atopic Dermatitis: A Rare Dermatological Convergence

**DOI:** 10.7759/cureus.62199

**Published:** 2024-06-11

**Authors:** Nouf AlJomah, Alaa AlShamsy, Ruba AlGarzai

**Affiliations:** 1 Dermatology, Armed Forces Hospital in Dharan, Dhahran, SAU; 2 Dermatology, Dammam Medical Complex, Dammam, SAU; 3 Dermatology, King Fahad Military Medical Complex, Dhahran, SAU

**Keywords:** eczema herpeticum, acute generalized exanthematous pustulosis, acute localized exanthematous pustulosis, alep, agep

## Abstract

Eczema herpeticum (EH) is a severe and potentially life-threatening viral infection occurring in individuals with preexisting eczema or atopic dermatitis. It is primarily caused by the herpes simplex virus, presenting as painful vesicular eruptions on the skin. On the other hand, acute localized exanthematous pustulosis (ALEP) is a rare variant of acute generalized exanthematous pustulosis (AGEP), characterized by the sudden onset of localized, nonfollicular pustules on an erythematous base. It is often triggered by recent medication administration, and its clinical presentation mimics AGEP, although ALEP exhibits a confined distribution of pustules. Prompt diagnosis and identification of the offending agent are crucial for effective management. Both are distinct cutaneous manifestations that rarely occur concurrently, presenting unique diagnostic and therapeutic challenges.

We present the first documented case of coexisting ALEP and EH in a 32-year-old male with a history of atopic dermatitis. The patient was admitted with features suggestive of EH, including vesicular lesions over the face, along with a positive Methicillin-resistant Staphylococcus aureus (MRSA) swab. Treatment with ceftaroline initially initiated resulted in the development of localized pustules, indicative of ALEP. Transition to linezolid led to the complete resolution of both conditions, marking a compelling recovery. The distinctive interplay between EH, ALEP, and AGEP presents a novel challenge, emphasizing the need for nuanced clinical assessment and tailored therapeutic strategies. This case offers crucial insights into the intricate relationship between medication-induced dermatological conditions and underlying cutaneous vulnerabilities. This unprecedented case highlights the rarity and complex management nuances associated with the simultaneous occurrence of ALEP and EH. The successful resolution following medication adjustments underscores the need for flexibility and comprehensive evaluation in addressing such intricate dermatological scenarios, providing valuable insights into potential synergies between distinct cutaneous conditions.

## Introduction

Eczema herpeticum (EH) is a disseminated cutaneous infection caused mainly by herpes simplex virus type I (HSV-I) [1]. It is a well-known complication observed in atopic dermatitis patients due to impairment of the epidermal barrier and immune dysregulation. Furthermore, EH is observed in other diseases such as Darier’s disease, epidermolysis bullosa simplex, and pemphigus foliaceus [1,2]. Acute localized exanthematous pustulosis (ALEP) is a variant of acute generalized exanthematous pustulosis (AGEP) but involving a localized area. It represents an unusual skin reaction that arises rapidly in the background of recent systemic drug administration. However, rare cases suggested infectious causes [3,4], while few cases reported ALEP secondary to topical treatment [5]. To our knowledge, this is the first reported case of a co-existence of ALEP and EH. Herein, we reported a 32-year-old male presented with ALEP on top of EH that resolved completely after discontinuation of the culprit drug.

## Case presentation

**Figure 1:**
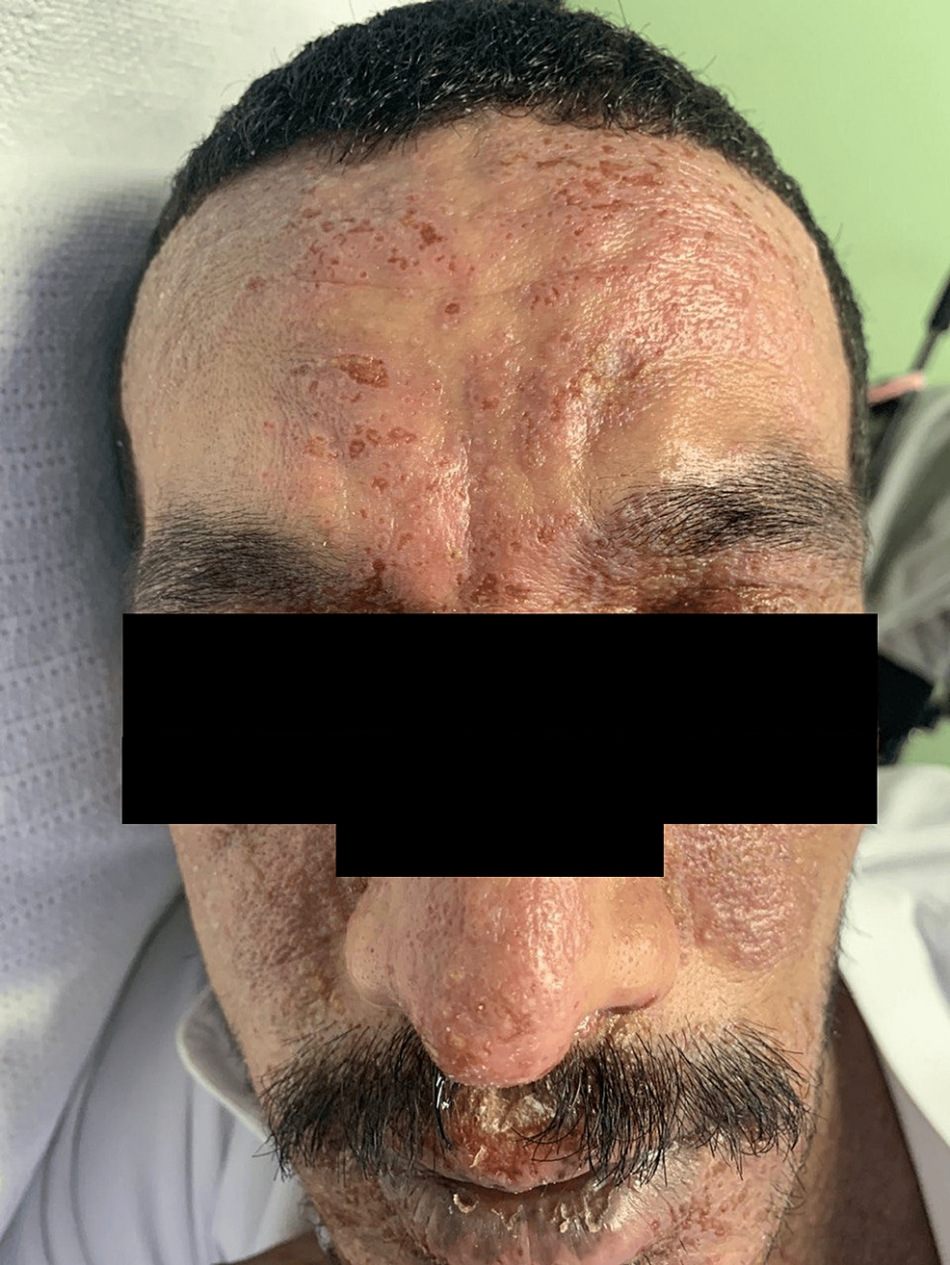
The condition of the patient upon presentation.

**Figure 2:**
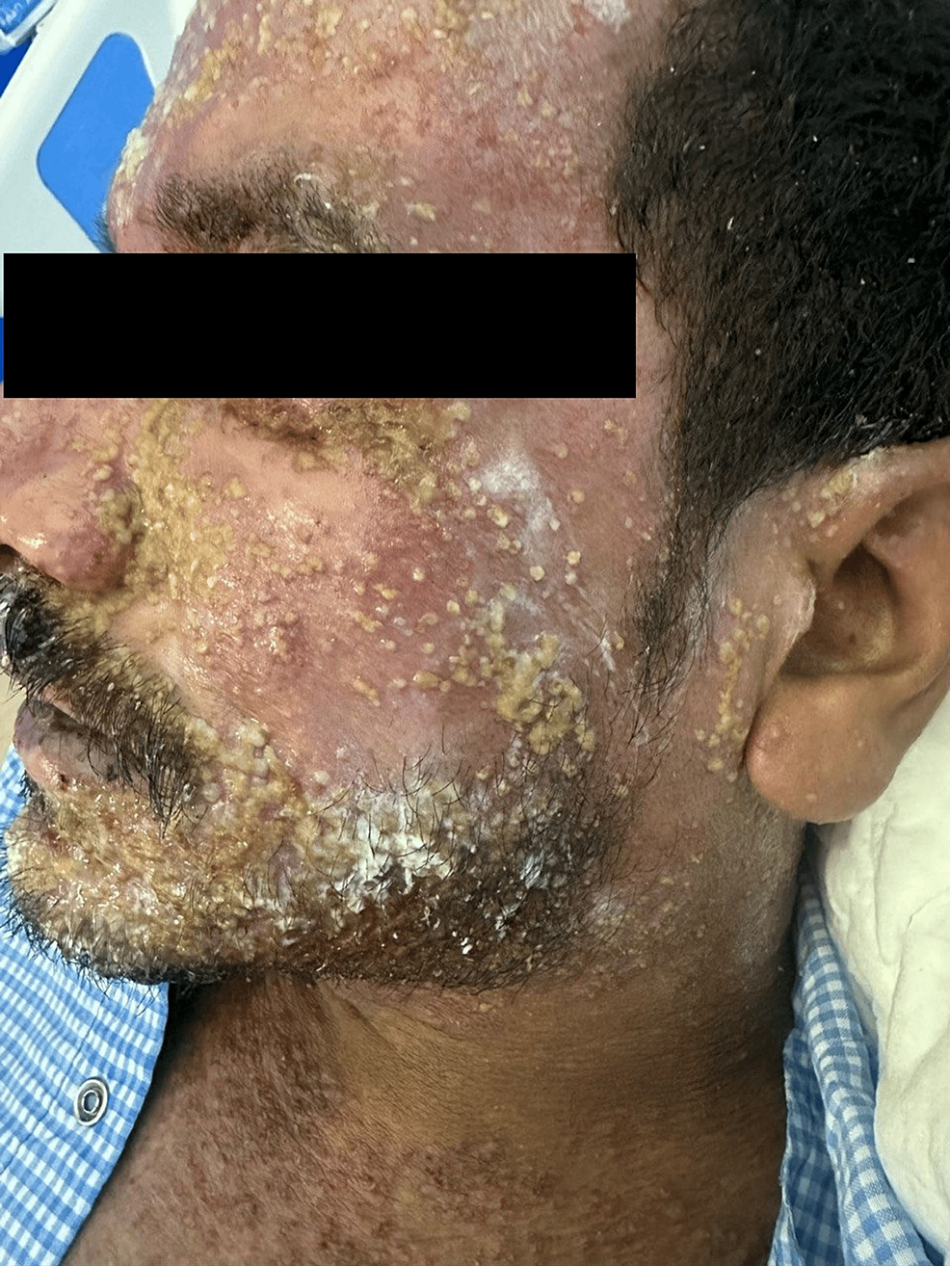
The condition of the patient two days after the initiation of ceftaroline.

**Figure 3:**
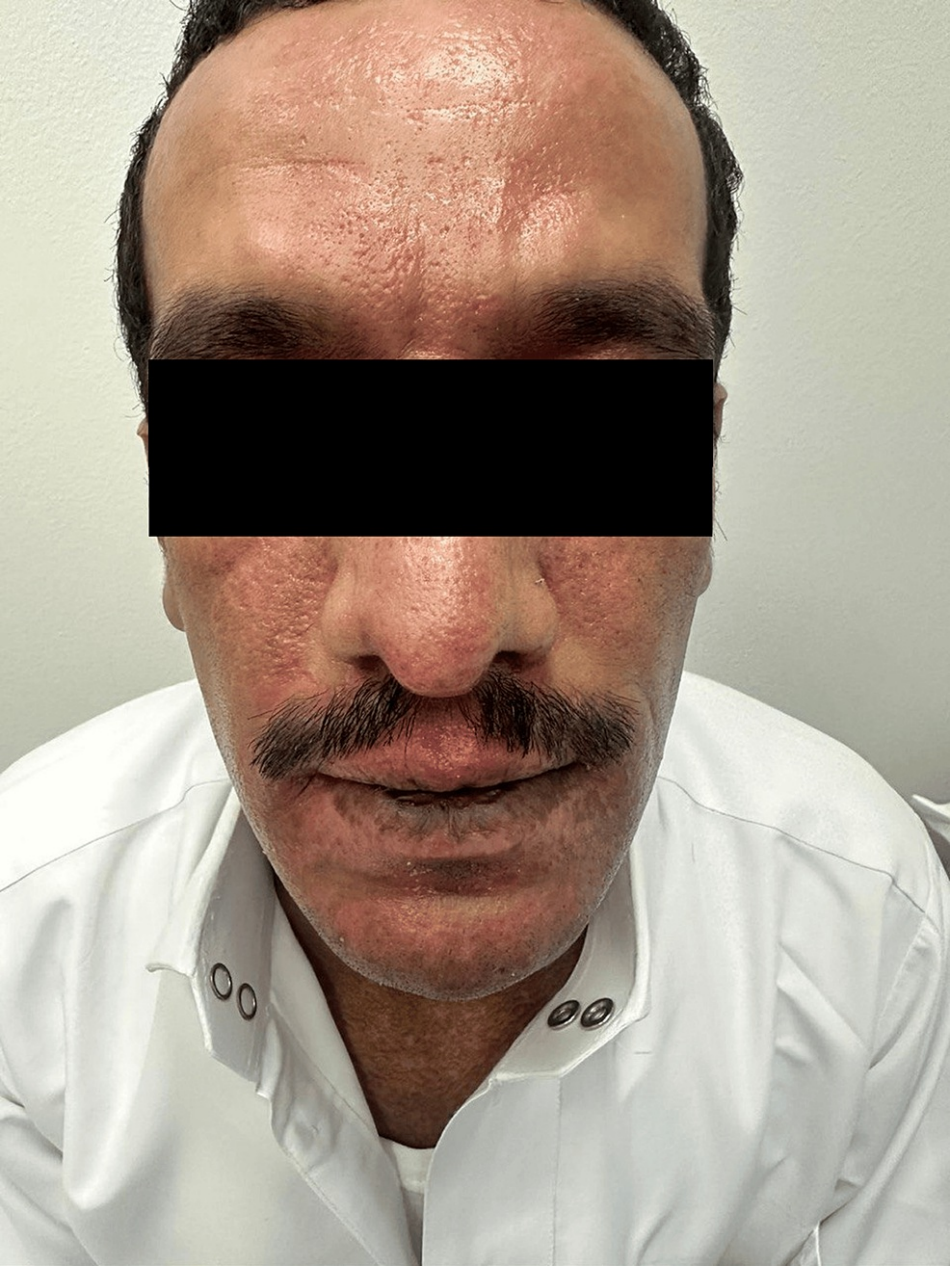
The condition of the patient one week after resolution of ALEP and two weeks since the beginning of EH. ALEP, acute localized exanthematous pustulosis; EH, eczema herpeticum

A 32-year-old male with a personal history of atopic dermatitis was referred from the Emergency Department (ED) at King Fahad Military Medical Complex with a complaint of painful skin lesions accompanied by fever for 3 days duration over the face and upper neck, associated with purulent eye discharge. Skin examination revealed multiple monomorphic vesicles with punched-out erosion over an erythematous background with hemorrhagic and yellowish crust over the face consistent with EH (Figure 1). Initial laboratory workup conducted by ED showed high C-reactive protein (CRP level 102.40, with a reference range of equal or less than 5) and a positive swab of MRSA. The patient was admitted under the internal medicine team, and a full septic workup, including swabs from vesicles, was sent. Acyclovir 10 mg/kg TID, ceftaroline 600 mg intravenous (IV) Q 8 hours, paracetamol 1 g IV three times daily along with skin-directed therapies (Fucidin, Mupriocin, Acyclovir), and topical eye drops were initiated. However, two days later, the patient suddenly developed a pustular eruption over the face; upon examination, we appreciated multiple nonfollicular pustules overlying an erythematous base (Figure 2). Based on clinical examination, a diagnosis of ALEP was established. A swab from the pustule was taken, ceftaroline was discontinued, and the patient was started on Linezolid 600 mg BID and given mometasone cream BID. The bacterial culture from the pus was sterile, and the CRP level normalized, reaching less than 1. The patient’s lesions started to improve with a complete resolution over a week from both conditions without any sequela (Figure 3).

## Discussion

AGEP is a rare and severe cutaneous adverse reaction characterized by a sudden onset of nonfollicular sterile pustules on an erythematous base, accompanied by systemic symptoms such as fever and leukocytosis. It is often drug-induced, with antibiotics and antifungal agents being common culprits examples include (β-lactams, clindamycin, and trimethoprim-sulfamethoxazole), nonsteroidal anti-inflammatory drugs (e.g., diclofenac and ibuprofen [5,6]. Other less likely causes are viral infections, ultraviolet radiation, and vaccination [6]. The diagnosis is based on clinical presentation, often supported by histopathological findings, and requires prompt identification and withdrawal of the offending agent [7,8].

Another condition worth noting is ALEP - a variant of AGEP characterized by the development of localized pustules without systemic symptoms. Around 40 cases were reported in the literature, most of them in females, and the lesions developed over the face [5,9]. Although sharing similarities with AGEP, ALEP differs in its localized nature, often attributed to a particular focus on skin irritation or contact dermatitis. Detailed clinical assessment is paramount in distinguishing ALEP from other pustular dermatoses, as prompt recognition aids in targeted management and potential prevention of recurrent episodes [10].

EH, on the other hand, represents a widespread cutaneous infection with the herpes simplex virus in patients with underlying eczema or atopic dermatitis. It manifests as painful, punched-out vesicles and pustules on an erythematous base, typically accompanied by fever, malaise, and lymphadenopathy. Prompt recognition and treatment are essential to prevent potential complications, including disseminated viral infection and systemic involvement [1].

The case of 32-year-old male, a known case of atopic dermatitis and lactulose intolerance, presented with fever and a rash involving the face. The initial diagnosis of EH was made, and a swab test confirmed the presence of MRSA. In response, the Infectious Disease (ID) team initiated treatment with ceftaroline. However, after two days, the patient developed multiple pustules over the face on a background of erythema.

The subsequent decision by the ID team to transition the patient from ceftaroline to linezolid resulted in a dramatic improvement in the patient's condition. This typical clinical picture with the negative test of the second swab result, in conjunction with the marked improvement in the patient's dermatological presentation, leads to a final diagnosis of a localized variant of AGEP. Notably, this represents the first reported case of ALEP associated with EH.

This case is unique due to the rare occurrence of AGEP in association with EH. Understanding this association is critical for expanding the knowledge base within the dermatological community, particularly in managing complex cases within this patient population.

The swift and striking response to linezolid following the initial adverse reaction to ceftaroline offers valuable insights into the nuanced management of medication-induced dermatologic conditions. This case represents a distinctive example of not only the association between EH and AGEP but also the pivotal role of appropriate medication selection in overcoming adverse treatment responses in complex dermatological scenarios.

Current literature primarily addresses EH and its complications but lacks substantial coverage of its association with localized variants of AGEP. The absence of documented cases similar to the one presented underscores the novelty of this finding and emphasizes the need for further evaluation and investigation.

The onset of pustules after ceftaroline administration accentuates the intricate relationship between medication reactions and preexisting dermatological conditions. This underscores the necessity of vigilance when treating patients with atopic dermatitis and a history of medication intolerances, particularly in light of the potential for atypical presentations.

## Conclusions

In conclusion, to our knowledge, this case represents the first published instance highlighting the co-occurrence of AGEP and EH. This case underlines the importance of remaining attentive to unusual presentations and treatment responses, particularly in patients with underlying atopic dermatitis. The rarity of this association emphasizes the need for continued vigilance and thorough investigation in the management of complex dermatological cases, shedding light on potential interrelations between seemingly distinct dermatological conditions.

The successful management of this case through the strategic adjustment of medication not only led to the patient's remarkable recovery but also shed light on the potential synergies between AGEP and EH. Finally, this case serves as a compelling testament to the intricacies of dermatological conditions and emphasizes the imperative of a meticulous and flexible approach in addressing such complex clinical scenarios.
